# Diesel soot coated non-woven fabric for oil-water separation and adsorption applications

**DOI:** 10.1038/s41598-019-44920-x

**Published:** 2019-06-11

**Authors:** Moolchand Sharma, Gurpreet Singh, Rahul Vaish

**Affiliations:** School of Engineering, Indian Institute of Technology Mandi, Mandi, Himachal Pradesh 175005 India

**Keywords:** Engineering, Materials science

## Abstract

The diesel soot (DS) coated non-woven fabric was studied for oil-water separation along with the adsorption of dyes, detergents, and pharmaceuticals. The DS coated non-woven fabric showed more than 95% separation efficiency and consistent repeatable performance during oil-water separation experiment. In addition to this, the DS coated non-woven fabric of 17.2 cm^2^ area successfully adsorbed ~85%, 97%, and 100% methylene blue (MB) dye, ciprofloxacin, and detergent, respectively from their respective solutions within 30 min, which was not possible using uncoated non-woven fabric. The DS coated non-woven fabric was found to be hydrophobic with the contact angle of 140° which was almost invariant upto 60 °C. Hence, the DS coated non-woven fabric showed promising performance in the oil-water separation and adsorption applications.

## Introduction

Increasing oil spill accidents and the generation of oily wastewater from industries is a major concern thesedays^1,2^.The water pollution caused due to the oil results in long lasting damage to the marine ecosystem^3^. The traditional oil-water separation methods such as floatation, filtration, distillation, use of oil skimmers, electrophoresis and centrifugation, etc. are time consuming and complicated^4,5^. Researchers are now focused on developing novel, simple, low cost, and highly efficient oil-water separation methods. In this direction, the special wettable materials possessing hydrophobic and oleophillic properties have emerged as promising method for oil-water separation^6,7^. By controlling the surface topology (surface chemistry and surface roughness), these materials can be made superhydrophobic and superoleophilic^7^. Zhang and Seeger grew silicone nanofilaments on polyester textile substrate possessing superhydrophobic/superoleophillic properties and showed the complete separation of the hexane oil from water^8^. Similarly, Zhang *et al*. fabricated multifunctional superhydrophobic and superoleophillic (MSHO) coating on stainless steel mesh, which provided the separation of various oils (hexane, toluene, kerosene, diesel, and chloroform) from water with separation efficiency of more than 93%^9^. Despite of the fact that various forms of special wettable materials such as foams, meshes, membranes, filter papers, etc. have been reported for promising oil-water separation efficiency, however, the complex chemical fabrication methods, poor recyclability, and degradation of efficiency with time and temperature poses limitation in the real-time applications^9–11^. Recently, the non-woven fabrics have gained attention in oil-water separation techniques^1^. The non-woven fabric is considered as a promising candidate for oil-water separation due to its light weight, low cost, flexibility, and resistant against corrosion^6^. Several other applications of the non-woven fabric include filtration of air, reclamation of domestic wastewater, and treatment of irrigation water etc^12^.

In addition to the oil, various organic dyes, detergents, and pharmaceuticals effluents from drug manufacturers pollute natural water sources such as river, lakes, etc^13,14^. Activated carbon has been widely investigated as an adsorbent for the adsorption of dyes, detergents, and pharmaceuticals^15^. Various other carbon family materials (graphene, CNT, etc.) have also been reported for water treatment applications through adsorption^16,17^. These alternatives cannot be utilized for large scale purposes due to their fabrication cost. Therefore, there is a need to explore other forms of carbon, which can be utilized for water cleaning and related applications. In this context, Singh *et al*. recently reported the adsorption of methylene blue and rhodamine B dyes through low cost and easily available porous candle soot containing carbon^18^. Similar to the candle soot, the another type of waste is diesel soot. Diesel powered vehicles emit significant amount of carbon monoxide (CO), nitrogen oxides (NO_X_) and the small fraction of soot particles^19,20^. These soot particles disturb the climate and contribute to global warming. Also, the soot particles are carcinogenic to human-beings, as these particles are inhaled by humans and get deposited in their lungs^21,22^. Hence, the soot particles are hazardous waste, which needs to be minimized. The minimization of diesel soot can be done using more efficient diesel engines and using exhaust after-treatment devices^23^. However, there exist still no method, that can bring down the diesel soot to zero. If this waste diesel soot cannot bring down to zero, then there is need to look for the applications, where it can be productively utilized. Clague *et al*. reported the fractions of compositional elements present in engine soot and exhaust soot provided by a diesel propelled vehicle and showed the presence of 80–90% and 40–50% carbon content in them, respectively^24^. Patel *et al*. provided the structural and compositional analysis of diesel soot using high resolution transmission electron microscopy (HR-TEM) and X-ray absorption near edge structure (XANES) spectroscopy techniques. The results showed the presence of turbostratic (graphitic) carbon structure region mainly along with the nano-particles of Ca_3_(PO_4_)_2_ and Fe_2_O_3_ embedded in turbostratic carbon structure^25^. The presence of turbostratic carbon structure in the waste diesel soot open up the gates for new applications of diesel soot by taking the advantage of properties of carbon element presents in the diesel soot. Similar to other members of carbon family, the diesel soot can also be used for water cleaning applications, which is not explored so far.

Herein, the coating of diesel soot on the non-woven fabric was provided, which can effectively be used for the adsorption dyes, detergents, and pharmaceuticals from the waste-water along with the oil-water separation. The low cost, easy fabrication, consistent water-separation efficiency, and promising adsorption properties are the advantages of diesel soot coated non-woven fabric over other reported materials.

## Results and Discussion

**Figure 1:**
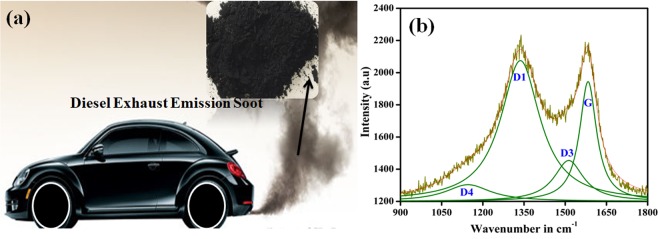
(**a**) Typical diesel powered car as a source of diesel exhaust emission soot (shown in inset) and (**b**) raman spectrum of diesel soot powder.

**Figure 2:**
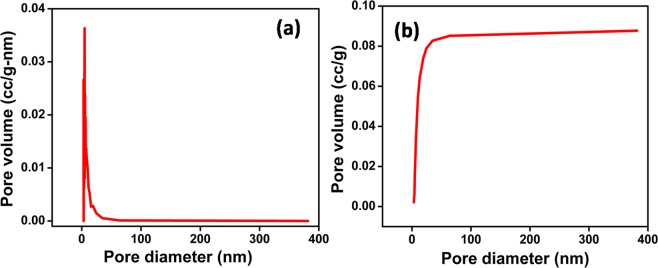
The pore size distribution of diesel soot in the (**a**) differential and (**b**) cumulative form.

Figure 1(a) shows a typical diesel powered car as a source of diesel exhaust emission soot. The diesel exhaust emission soot was obtained in powder form from the vehicle exhaust pipe. Figure 1(b) shows the Raman spectrum obtained for DS powder in order to know the structure of carbons present in the diesel soot. The analysis of Raman spectrum was done by peak fitting method using Lorentzian function. After fitting, the four peaks were observed at ~1146, ~1336, ~1512 and ~158  cm^−1^ wavenumbers. The band presents at ~158  cm^−1^ (known as G-band) could be assigned to vibrational mode of ideal graphite lattice having E_2g_ symmetry^22,26^. The band at ~158  cm^−1^ (known as D1-band) was actually a characteristic of disordered graphite lattice. D1-band (possessing A_1g_ symmetry) could be assigned to vibrational mode of disordered graphite lattice mainly at the edges of graphene layers. The band at ~151  cm^−1^ (known as D3-band) was observed due to presence of fraction of amorphous carbon, which consisted of organic molecules, functional groups, and sp^2^ bonded forms of carbon. The band at ~114  cm^−1^ (known as D4-band) was found due to disorder produced by the presence of polyenes and ionic impurities. Thus, Raman spectrum showed the presence of graphite form of carbon presents in the diesel soot along with disorderness due to the presence of functional groups, ionic impurities, and amorphous carbon. The $\frac{G}{D}$ ratio (where, *G* is the intensity of G-band and D is the intensity of sum of D1, D3, and D4 bands) provides the ratio of graphite to the disordered graphite content in the diesel soot^27,28^. More detailed information can be extracted by examining the contributions from different disordered forms of carbon, i.e. by evaluating $\frac{{\rm{G}}}{{\rm{D}}1}$, $\frac{{\rm{G}}}{{\rm{D}}3}$, and $\frac{{\rm{G}}}{{\rm{D}}4}$ ratios. The values of $\frac{G}{D}$, $\frac{{\rm{G}}}{{\rm{D}}1}$, $\frac{{\rm{G}}}{{\rm{D}}1}$, $\frac{{\rm{G}}}{{\rm{D}}3}$, and $\frac{{\rm{G}}}{{\rm{D}}4}$ are provided in Table 1. These calculated values was found to be higher than the values achieved in the case of carbon black and diesel soot extracted from vehicle test, engine dynamometer test and exhaust provided in the reference^27^. Higher value of $\frac{G}{D1}$ showed the smaller proportion of the disordered edges of graphene layers. Higher value of $\frac{G}{D3}$ showed the smaller fraction of amorphous carbon. The higher value of $\frac{{\rm{G}}}{{\rm{D}}4}$ showed the smaller proportion of presence of polyenes and ionic impurities. This clearly shows that the smaller proportion of disorderness was present in case of DS used in the present study^27^. Han *et. al* reported the increase in hydrophobicity with the increase in $\frac{G}{D}$ ratio^29^. Thus, the less disorderness helps in achieving higher hydrophobicity. The differential and cumulative pore size distributions are shown in Fig. 2(a,b) respectively, which clearly show that the pore size lies in the range of 4–50 nm. The average pore size was found to be 4.75 nm.

**Table 1:** Raman spectroscopic data for diesel soot.

Peak Designation	Peak position	Full width at half maximum(FWHM)	Intensity	$\frac{{\bf{G}}}{{\bf{D}}{\bf{1}}}$	$\frac{{\bf{G}}}{{\bf{D}}{\bf{3}}}$	$\frac{{\bf{G}}}{{\bf{D4}}}$	$\frac{{\bf{G}}}{{\bf{D}}{\bf{1}}{\boldsymbol{+}}{\bf{D}}{\bf{3}}{\boldsymbol{+}}{\bf{D}}{\bf{4}}}{\boldsymbol{=}}\frac{{\bf{G}}}{{\bf{D}}}$
**D4**	1146.21	198.30	129.63			7.19	0.60
**D1**	1336.67	173.45	1095.93	0.85			
**D3**	1512.77	129.42	317.86		2.93		
**G**	1583.12	74.88	932.22

**Figure 3:**
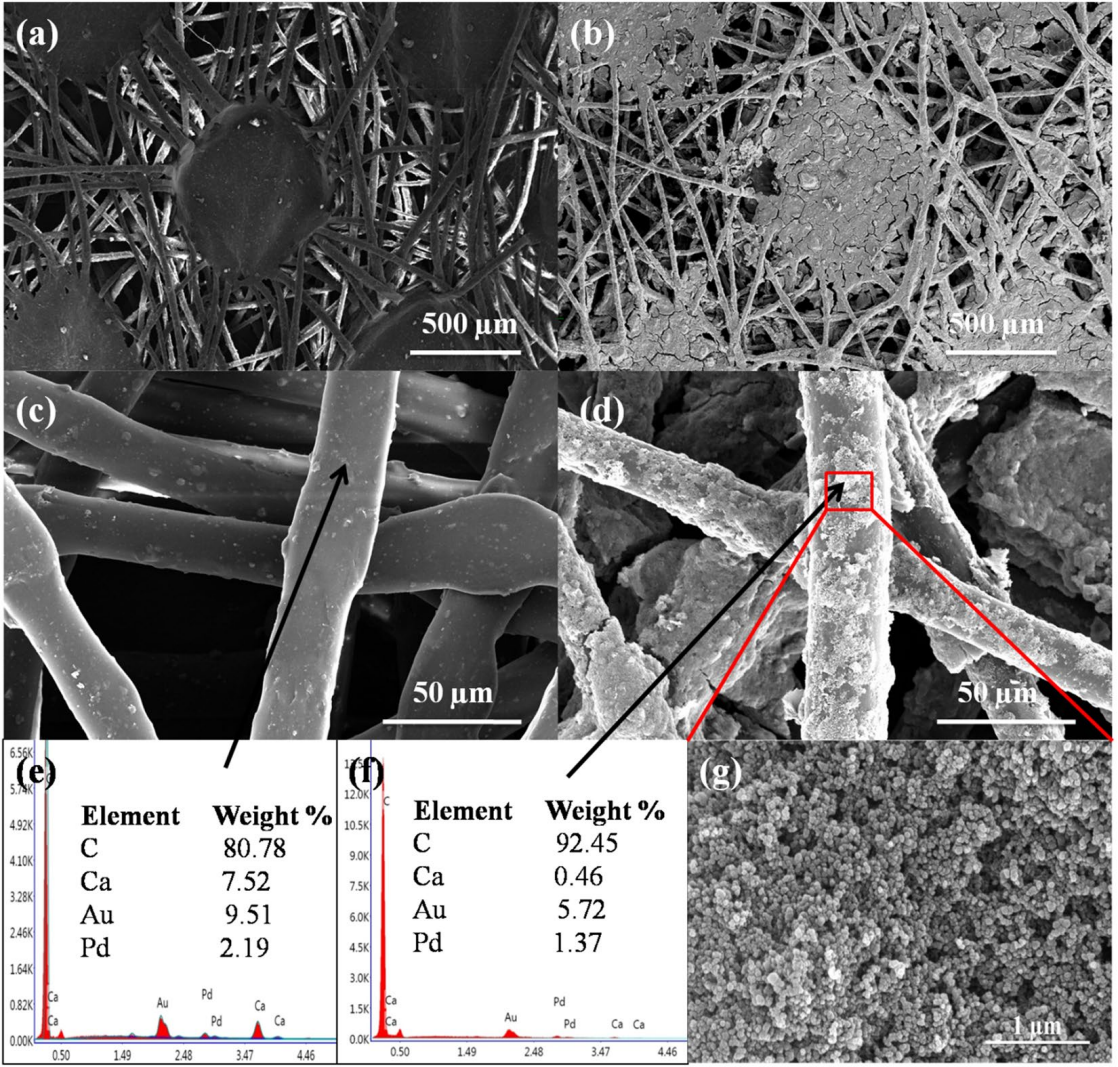
SEM micrographs of (**a,c**) uncoated non-woven fabric, (**b,d**) DS coated non-woven fabric, (**e,f**) EDS spectra of uncoated and DS coated fabric, respectively, and (**g**) SEM micrograph of DS particles stick to the fabric of DS coated non-woven fabric.

Figure 3 shows SEM micrographs of uncoated and DS coated non-woven fabrics. It can be easily seen in the Fig. 3(a–d) that both the uncoated and DS coated non-woven fabrics had porous structure. Moreover after DS coating, diesel soot particles stick along fibres of non-woven fabric as shown in the Fig. 3(d). EDS spectrum of uncoated fabric clearly showed that the chemical composition of non-woven fabric mainly consisted of carbon and calcium elements as shown in Fig. 3(e). The calcium carbonate filler is generally added in the non-woven fabrics in order to improve the strength, stiffness, opacity, whiteness, glossiness and eliminates water carrying, due to which Ca element was observed in EDS of non-woven fabric. EDS spectrum of diesel soot (powder) showed the presence of carbon, iron, oxygen, aluminum, sulphur elements (Supplementary Fig. 2). However, in DS coated fabric, diesel soot particles stick along the fibres mainly consisted of carbon only as shown in the Fig. 3(f). Moreover DS particles stick to fibres possessed porous structure formed by agglomeration of nano-sized particles as shown in the Fig. 3(g).

**Figure 4:**
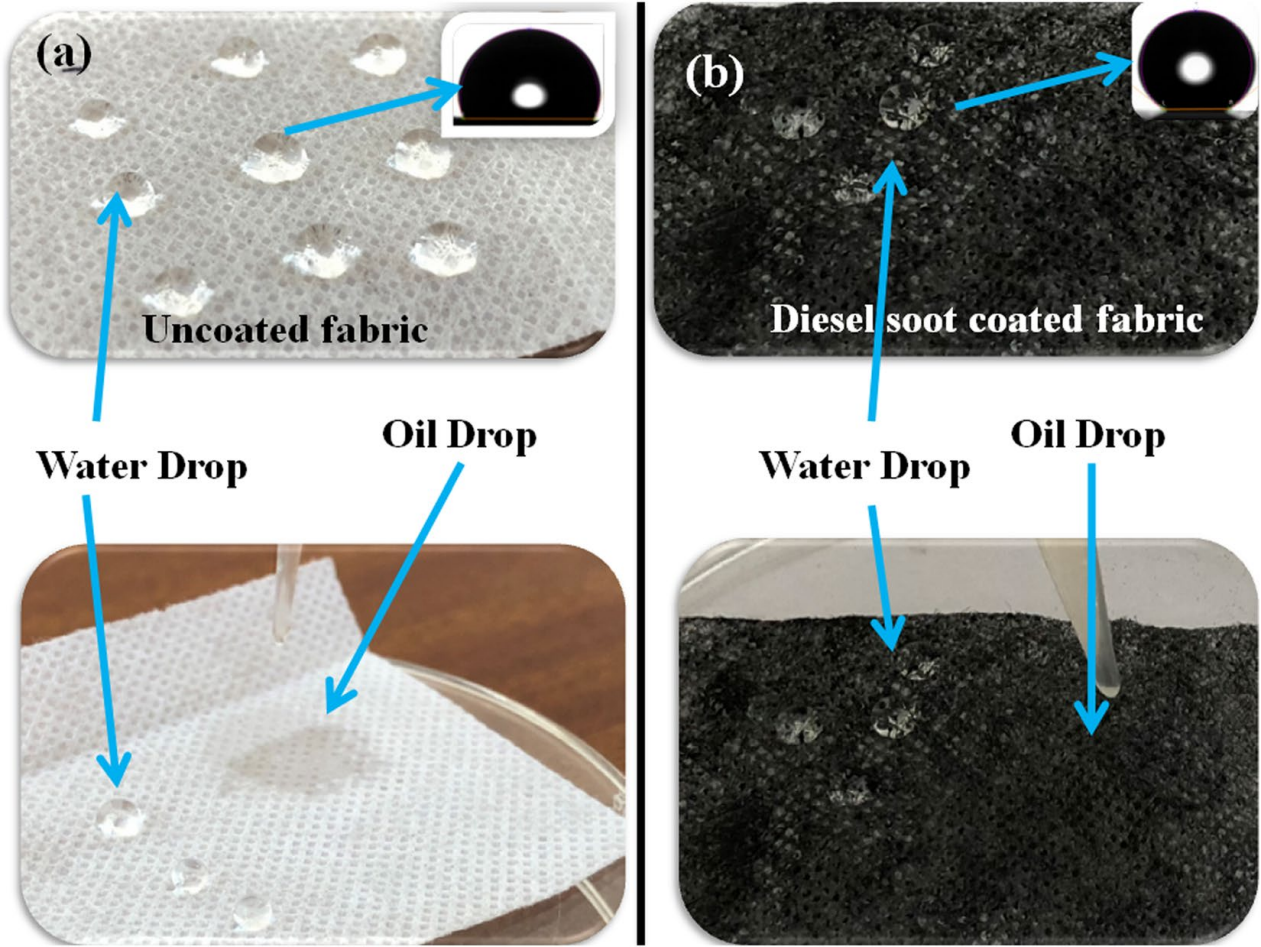
Hydrophobic and oleophilic nature of (**a**) uncoated, and (**b**) DS coated non-woven fabrics.

Figure 4 shows that uncoated and DS coated non-woven fabrics can be used as an oil–water separator. When the five water drops were dropped down on uncoated and DS coated non-woven fabrics, the drops stayed on each surface and the mean values of contact angles of the water drops were found to be 105.4° and 140.2° with the error of ±2.1° and ±2.8°, respectively. This clearly showed the hydrophobic nature of both uncoated and DS coated non-woven fabrics. However, the hydrophobicity of DS coated non-woven fabric was more than that of the uncoated non-woven fabric. Both the surface chemistry and the surface roughness factors are playing the roles. The graphene layers of diesel soot possessed C=O, and C–H functional groups^30^. It is already known that C–H groups are hydrophobic in nature and C=O are hydrophilic in nature^29^. Out of $\frac{{\rm{G}}}{{\rm{D}}1}$, $\frac{{\rm{G}}}{{\rm{D}}2}$, and $\frac{{\rm{G}}}{{\rm{D}}3}\,$, the $\frac{{\rm{G}}}{{\rm{D}}3}$ value is lower, which means that the defects are mainly present at graphene layer edges. These defects possessed mainly hydrophilic C=O groups, however the graphene layer surfaces possessed mainly hydrophobic C–H groups. Thus, less disorderness at graphene layer surface contributed for hydrophobicity of diesel soot^29^. Thus, the hydrophobic nature of diesel soot coating is the one major factor for higher hydrophobicity in case of DS coated non-woven fabric. In addition to this, the Fig. 3(c,d) clearly show the more rough surface of DS coated non-woven fabric than that of the uncoated non-woven fabric. Also it is well known that, the increase in surface roughness led to increase in hydrophobicity^11^. Thus, the increased surface roughness of the non-woven fabric with DS coating also contributed to increased hydrophobicity.

On the other hand, when the oil (petrol) drops were dropped down on uncoated and DS coated non-woven fabrics, the drops were not able to stay on the surface and passed through the fabric. Due to this reason, the contact angles could not recorded for oil drops. However, this clearly showed the oleophilic nature of both uncoated and DS coated non-woven fabrics. Both uncoated and DS coated fabrics can be easily used as an oil–water separator due to their hydrophobic and oleophilic nature. The oil (petrol)-water separation using uncoated and DS coated fabrics can be seen in Supplementary videos V1, V2.

**Figure 5:**
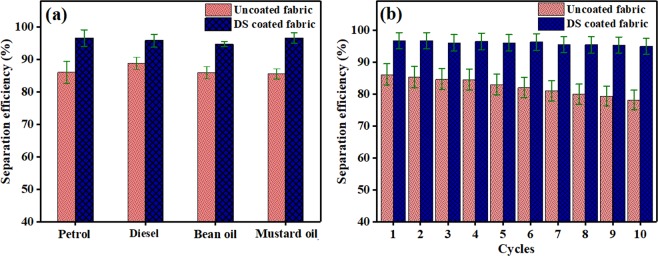
(**a**) Separation efficiencies of the uncoated and DS coated fabrics for various oil -water mixtures and (**b**) separation efficiency of the uncoated and DS coated fabrics with petrol-water mixture during repeated ten cycles of experiment.

Figure 5(a) shows separation efficiencies of uncoated and DS coated fabrics with various oil-water mixtures. The DS coated fabric showed more separation efficiencies than that of the uncoated non-woven fabric. This was due to increased hydrophobicity in case of the DS coated non-woven fabric than that of the uncoated fabrics. Figure 5(b) shows negligible change in separation efficiency of the DS coated non-woven fabric even after 10th cycle of petrol-water separation. However, the separation efficiency of uncoated fabric decreased with repeated number of cycles. This clearly indicated the promising repeatability of DS coated fabric over uncoated fabric.

**Figure 6:**
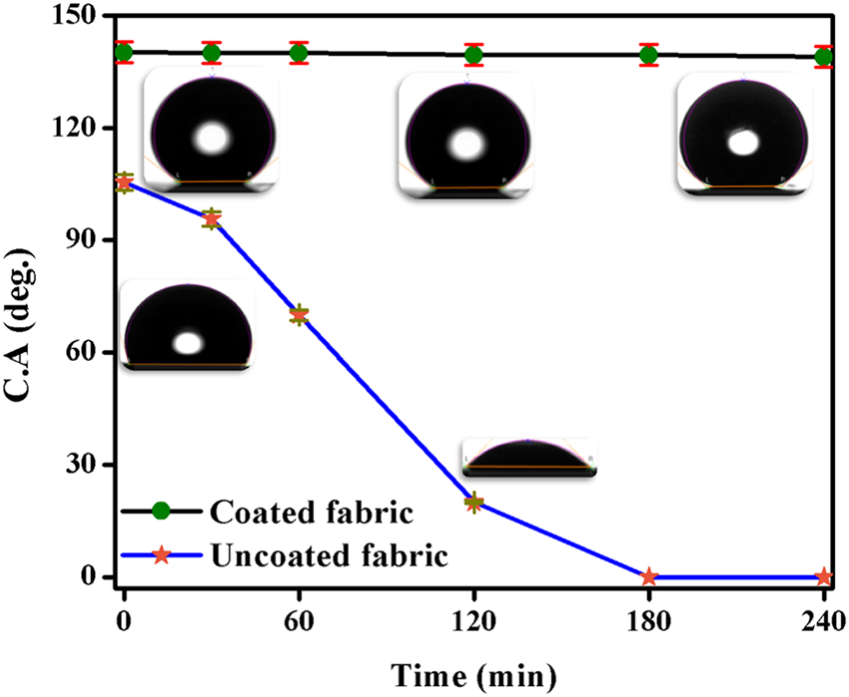
Variation of water contact angle with time for uncoated and DS coated non-woven fabrics.

In order to assess the effect of durability of uncoated and DS coated non-woven fabrics as a oil-water separator, the water contact angles of uncoated and DS coated non-woven fabric with respect to time were recorded as shown in the Fig. 6. In case of uncoated non-woven fabric, initial water contact angle of 105.4° ± 2.1° was observed, however this drop was completely passed through uncoated fabric after 3 h. On the other hand, DS coated non-woven fabric maintained its water contact angle of 140.2° ± 2.8° even after 4 h. This clearly showed that the DS coated non-woven fabric will be more durable as an oil–water separator as compared to the uncoated non-woven fabric.

**Figure 7:**
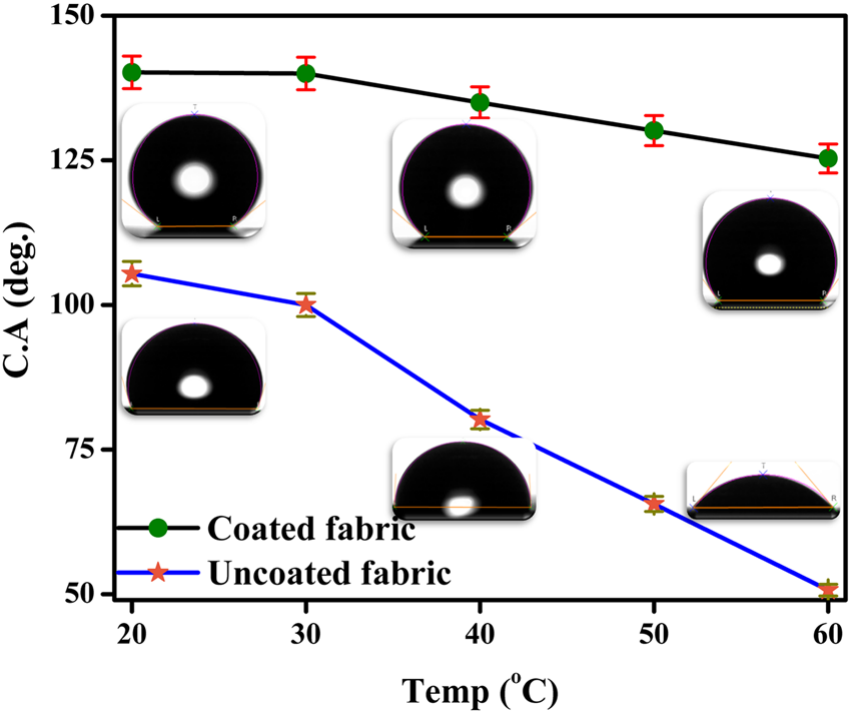
Variation of water contact angle with temperature in case of uncoated and DS coated non-woven fabric.

Figure 7. displays the variation of water contact angle with temperature for uncoated and DS coated non-woven fabrics. Figure 7 clearly showed that DS coated non-woven fabric has a small decrease in water contact angle (140.2° ± 2.8° to 125.32° ± 2.5°) with increase in temperature from 20 to 60 °C and maintained its hydrophobic nature. On the other hand, uncoated non-woven fabric showed hydrophobic to hydrophilic conversion with increase in temperature from 20 to 60 °C (contact angle decreased from 105.4° ± 2.1° to 50.7° ± 1.0°). This clearly showed that the DS coated non-woven fabric can be used as an oil–water separator even at 60 °C.

**Figure 8:**
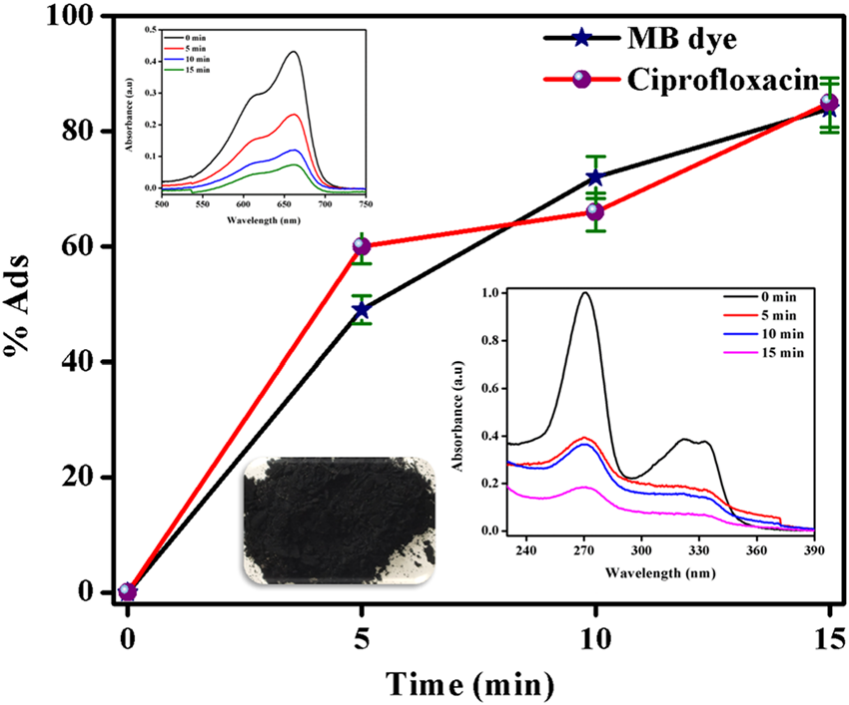
Adsorption of MB dye and ciprofloxacin with time using diesel soot powder. Insets shows the absorbance vs wavelength plots obtained during adsorption MB dye (left) and ciprofloxacin (right) using diesel soot powder.

In order to further study, the adsorption studies were performed. Figure 8 displays adsorption of MB dye and ciprofloxacin with time using diesel soot powder. Figure 8 clearly shows that 20 mg of DS powder adsorb 85 ± 2.5% and 87 ± 2.7% of MB dye and ciprofloxacin from their respective solutions of 22 mg/L and 15 mg/L initial concentrations, respectively, within 15 min. The decrement in absorbance maximum peak of MB dye (at 664 nm) and ciprofloxacin (at 275 nm) with time can be easily seen in the inset of Fig. 8. Hence, DS powder was found to be a promising adsorbent for adsorption of MB dye and ciprofloxacin. The adsorption capacity of DS powder was mainly due to its porous structure. The porous structure of DS can be easily seen in the Fig. 3(g). The porous structure provided sites for the adsorption of dyes and pharmaceuticals. Moreover, Alrefaai *et al*. reported the functional groups present in the diesel soot identified using Fourier transform infrared (FTIR) spectroscopy^30^. Four main peaks (high intensity peaks) were shown in the FTIR. These were, 1) C–H stretching modes for the aromatic (3000 cm^−1^) and aliphatic (2950, 2925 and 2860 cm^−1^) groups, 2) C=C stretching of aromatics at 1600 cm^−1^, 3) C=O stretching vibration (at 1725 cm^−1^) present in various aldehydes, carboxylic acid, esters, anhydrides, and ketones, and 4) –OH stretching modes (at 3300 and 3650 cm^−1^), and it arises from different carbon sources such as carboxylic acid and phenolic groups^30^. Based on the functional groups, four possible mechanisms can be provided for the adsorption of MB dye on the diesel soot. These are: (1) the negative charge on carboxylate anion (COO) electrostatically interact with the positive charge present on N atom of MB dye, (2) the hydrogen bond formation between H atom (from –OH group) and N atom (from MB dye), (3) the carbonyl group donates electron to aromatic ring of MB dye. (4) *π*-*π* interaction between aromatic ring of MB dye and the aromatic structure of graphene layer^31^.

**Figure 9:**
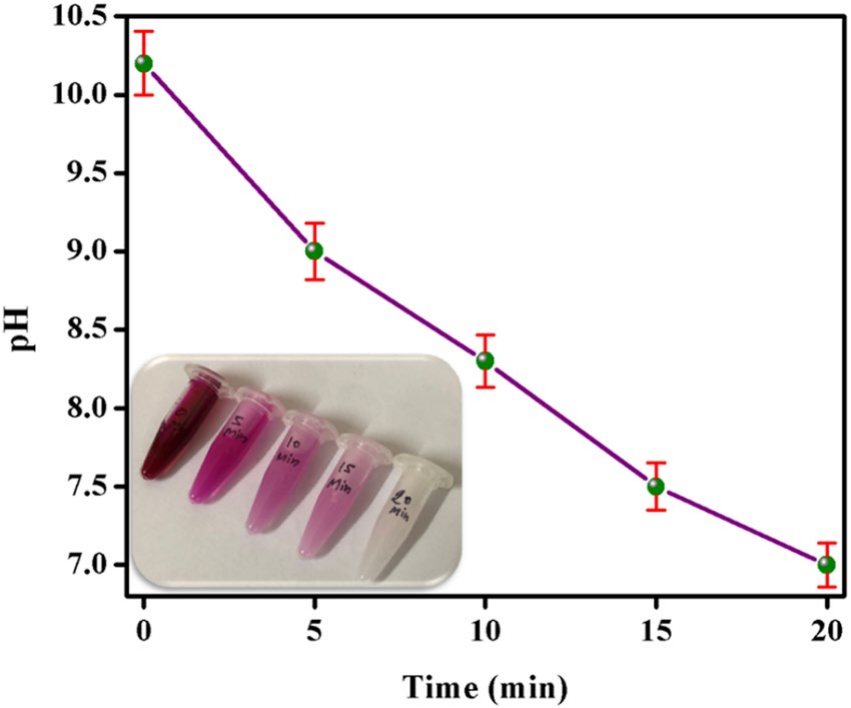
Variation in pH of detergent solution with time during adsorption of detergent using DS powder. The inset shows the change in colour of detergent solution containing phenolphthalein indicator with time during adsorption experiment.

DS (powder) was also found to be good adsorbent for detergent also. The prepared detergent solution of 2.5 mg/L initial concentration possessed alkaline pH value of 10 ± 0.2. During adsorption experiment using 20 mg of DS powder, the pH of solution started decreasing with time. After 20 min. of adsorption experiment, pH value reduced to ~7 (neutral) as shown in Fig. 9. The decrease in pH value upto 7 ± 0.14 clearly indicated the removal of detergent from the detergent solution. As detergent solution generally showed no colour, so phenolphthalein was added as a colour indicator. The detergent water showed dark pink colour with phenolphthalein initially. However, during adsorption of detergent, the dark pink colour started changing to light pink and finally got colourless after 20 min. The change in dark pink colour can be easily seen in the inset of Fig. 9. The colour change is also an another indicator of removal of detergent from the solution. Therefore, DS powder could be a promising adsorbent for dyes, pharmaceuticals, and detergents.

**Figure 10:**
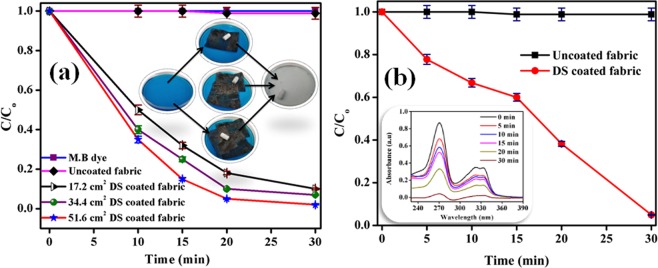
$\frac{C}{{C}_{o}}$ vs time plots during adsorption of (**a**) *Methylene blue* (MB) pollutant dye, and (**b**) ciprofloxacin using uncoated and DS coated non-woven fabrics. The inset of (**a**) shows the decolorization of dye solution during adsorption experiment. The inset of (**b**) shows the absorbance vs wavelength plot obtained during adsorption of ciprofloxacin using DS coated non-woven fabric.

It is to be noted that the adsorption through DS powder is not a practical solution because of some limitations such as agglomeration of powder particles, difficult to recover the powder after experiment etc. However, these limitations can be easily removed by providing DS coating on a surface. Hence, the adsorption through DS coated non-woven fabric could be a possible practical solution. Figure 10(a) shows $\frac{{\rm{C}}}{{{\rm{C}}}_{{\rm{o}}}}$ vs time plots during adsorption of *Methylene blue* (MB) pollutant dye adsorbate by using uncoated and DS non-woven fabric (where C is the concentration of dye at any time ‘t’, and C_o_ is the initial dye concentration). The decrease in $\frac{{\rm{C}}}{{{\rm{C}}}_{{\rm{o}}}}$ is an indicator of decrease in concentration of dye in the dye solution, and hence got clean water free from dye. It can be easily seen in the Fig. 10 that there was negligible decrease in $\frac{{\rm{C}}}{{{\rm{C}}}_{{\rm{o}}}}$ ratio in case of uncoated non-woven fabric. On the other hand, in case of the DS coated non-woven fabric (using one layer of fabric and possessed 32 mg of sticked DS particles), the $\frac{{\rm{C}}}{{{\rm{C}}}_{{\rm{o}}}}$ ratio decreased from 1 to 0.15 ± 0.005 within 30 min during adsorption of MB dye from the solution of 22 mg/L initial concentration. Alongwith this, the decrease in blue colour of MB dye solution with time during adsorption experiment can be easily seen in Fig. 10(a). Hence, the DS coated non-woven fabric can be considered as a promising candidate for absorption of coloured dyes present in waste water coming from textile industries. The adsorption capacity of DS non-woven fabric was mainly due to the presence of diesel soot on fabric, which had porous structure^26,32^. These porous structure provided sites for the adsorption of dye. The value of $\frac{{\rm{C}}}{{{\rm{C}}}_{{\rm{o}}}}$ decreased from 1 to 0.15 ± 0.005, 0.1 ± 0.0035, and 0.02 ± 0.001 within 30 min during adsorption of MB dye by using one, two, and three layers of DS non-woven fabric, respectively. This shows that the rate of adsorption was increased with increase in number of adsorbent layers due to increase in surface area. In addition to this, Fig. 10(b) shows that DS coated non-woven fabric was also able to adsorb ciprofloxacin, however uncoated non-woven fabric showed no adsorption of ciprofloxacin. The value of $\frac{{\rm{C}}}{{{\rm{C}}}_{{\rm{o}}}}$ decreased from 1 to 0.03 ± 0.001 within 30 min during adsorption of ciprofloxacin using one layer of DS non-woven fabric. Therefore, DS coated non-woven fabric possessed promising adsorption capacity for pharmaceuticals waste.

**Figure 11:**
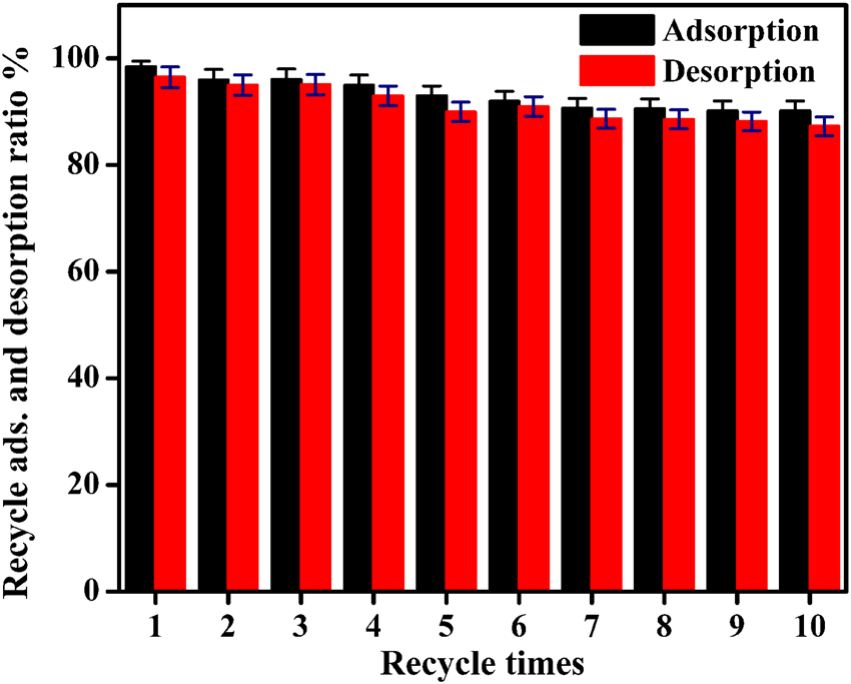
Repeated performance of DS non-woven fabric (three layers) during adsorption-desorption of *Methylene blue* (MB) dye.

Figure 11 shows the repeated performance of DS non-woven fabric (three layers) during adsorption-desorption of *Methylene blue* (MB) pollutant dye. The adsorption of MB dye using DS non-woven fabric (three layers) was found to be 90 ± 1.8% after 10th cycle, which was 98 ± 1% after first cycle. It showed that there was no severe change found in adsorption capacity of DS non-woven fabric (three layers) even after 10 cycles. This clearly shows the consistent performance of DS non-woven fabric in the adsorption of MB dye.

**Figure 12:**
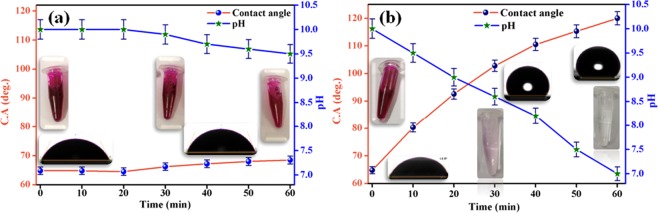
Variation of contact angle and pH value of detergent solution with time during adsorption experiment using (**a**) uncoated, and (**b**) DS coated non-woven fabrics.

DS coated non-woven fabric can also be used for adsorption of detergent from detergent solution. However, uncoated non-woven fabric was not able to adsorb detergent. The detergent solution having pH of 10 ± 0.2 showed the contact angle of 65 ± 1.2 on both uncoated and DS coated non-woven fabric initially. During adsorption experiment, no significant change in pH value and contact angle was observed in the case of uncoated non-woven fabric as shown in Fig. 12(a). This clearly indicated that uncoated non-woven fabric was not able to adsorb detergent from the solution.

However, the pH value of detergent solution started decreasing and contact angle started increasing with time in case of DS coated non-woven fabric. The decrease in pH value and increase in contact angle is a clear indicator of decrease in concentration of detergent in the solution. The value of pH of detergent solution of 2.5 mg/L initial concentration decreased from 10 ± 0.2 to 7 ± 0.14 and contact angle increased from 65 ± 1.2 to 118 ± 1.4 within 60 min DS coated non-woven fabric possessed 32 mg of sticked DS particles (as shown in Fig. 12(b). The pH value of ~7 and hydrophobic angle clearly indicated that all the detergent had been removed from the solution.

The removal of detergent is also shown by using phenolphthalein colour indicator. During adsorption of detergent using DS coated non-woven fabric, the dark pink colour changed to light pink and finally got colourless as shown in Fig. 12(b). However, no change in dark pink colour was observed in case of uncoated non-woven fabric as shown in Fig. 12(a). The colour change is also an indicator of removal of detergent of solution. Therefore, DS coated non-woven fabric has promising adsorption capacity for detergent.

In comparison to other nanoparticle and polymer coated hydrophobic surfaces mentioned in literature^33,34^, the present DS coated non-woven fabric provides a low-cost and easy to implement oil-water separation technique. Moreover, the separation efficiencies of present DS coated non-woven fabric are comparable to nanoparticle and polymer coated surfaces. Unlike nanoparticle and polymer coated surfaces, DS coated non-woven fabric also showed good adsorbent characteristics.

## Conclusions

Oil-water was successfully separated by using diesel soot coated non-woven fabric. These coated fabrics have also demonstrated repeated adsorption of various organic pollutants such as dyes, detergents, and pharmaceuticals. These fabrics exhibited hydrophobic characteristics with the contact angle of 140°. Hydrophobicity was stable upto 60 °C and invariant in time duration under study unlike the uncoated non-woven fabric. The low cost, easy fabrication, superior performance, and promising durability make the diesel soot coated non-woven fabric a promising alternative to already existing materials in oil-water separation and adsorption applications. However, the present work is only a laboratory scale testing and the detailed real-time investigation still needs to perform considering temperature, relative humidity etc. variants.

## Experimental

Diesel soot (DS) was collected from the exhaust tailpipe of a diesel powered vehicle. The collected diesel soot was used directly without undergoing any further purification process. The spunbound polypropylene non-woven fabric (thickness of 0.05 mm) was used in the present work, which is used as carry bag in our daily life. DS coated non-woven fabric was obtained by using dip coating process. For this, a dispersion of diesel soot in acetone medium was prepared by stirring using magnetic stirrer at 80 °C. A non-woven fabric of weight 0.1 g was dipped in diesel soot-acetone dispersion for 15 min, due to which a coating of diesel soot was obtained on non-woven fabric as shown in Supplementary Fig. 1. DS coated non-woven fabric was dried in an oven at 75 °C for 1 h. After drying, DS coated non-woven fabric was ultrasonically cleaned in acetone medium many times until no diesel soot particle came out of fabric into solution. The weight of DS coated non-woven fabric was found to 0.132 g, which indicated that 0.032 g soot particles got deposited on fabrics.

DS (powder) was characterized under Raman spectroscopy. Raman spectroscopy was performed on diesel soot powder using HORIBA (Model-Lab RAM HR Evolution). DS powder was excited by using green laser beam having wavelength of 535 nm with 100% power. Raman spectrum was obtained in the wavenumber range of 900–1800 cm^−1^. The Brunauer–Emmett–Teller (BET) method was used to measure the pore size of diesel soot using an Autosorb iQ Station 2 (Quantachrome Instruments, USA) with N_2_ at 77 K. The uncoated and DS coated non-woven fabric were observed under scanning electron microscope (FE-SEM Inspect^TM^S50) in order to visualize the microstructure. For this, a sample of 1 × 1 cm^2^ was cut from each uncoated as well as DS coated fabric. As these samples were non-conducting samples, then a 5 nm coating of gold-palladium (Au-Pd) was provided on each sample in order to observe under SEM. Energy dispersive spectroscopy (EDS) facility was used to identify the chemical composition of uncoated and DS coated non-woven fabric.

In order to examine the behaviour of uncoated and DS coated non-woven fabric towards water and oil at room temperature, the water and oil drops of 100 *μ*l were poured on uncoated and DS coated non-woven fabrics. Five drops of each (water and oil) were placed on five different locations on both (uncoated and DS coated) fabrics. The contact angles of drops were obtained by using contact angle meter (Kyowa Interface Science Co. Ltd., Japan). The detailed information about contact angle meter is provided in Supplementary Fig. 3 and text next to it. Then, the five water and five contact angles were measured for each fabric. The mean and the standard deviation (error) values of the five readings were determined. The contact angles of drops were obtained with respect to time (0 to 240 min.) and temperature (20 to 60 °C). The mean and the standard deviation (error) values were determined in the similar manner as discussed for contact angles measurements at room temperature.

The oil-water separation was done for four different oil-water mixtures (petrol-water, diesel-water, bean oil-water, and mustard oil-water). The known properties of oils such as densities and viscosities are presented in Supplementary Table 1. In oil-water separation experiment, 20 ml of water and 20 ml of oil were mixed in a beaker. The weight of oil before oil-water separation was noted down. Then, two more beakers were taken. The top of one beaker was covered with uncoated non-woven fabric and the top of other beaker was covered with DS coated non-woven fabric. The mixture of oil and water was poured on each beaker, so that oil-water separation can take place. See Supplementary videos V1, V2 for more clearlity. After oil-water separation, the mass of oil after separation was noted down. The experiment was repeated for five times in order to get mean and standard deviation values for each oil. The separation efficiency of uncoated and DS coated non-woven fabric was calculated as^9^:

1 $${\rm{Separation}}\,{\rm{efficiency}}=\frac{{m}_{a}}{{m}_{o}}\times 100$$

where, *m*_*a*_ is the mass of oil after separation, and *m*_*o*_ is the mass of oil before separation.

In order to investigate the adsorption capacity of DS powder, uncoated and DS coated non-woven fabric, adsorption experiment was performed by using *Methylene blue* (MB) dye, ciprofloxacin (pharamaceutical), and Surf excel detergent as adsorbates. MB dye is a commonly found pollutant in waste-water coming from textile industries^35^. MB dye is a cationic dye whose chemical formula is C_16_H_18_ClN_3_S^36^. Ciprofloxacin is a synthetic antibiotic, which released into water sources as effluents coming from drug manufacturers^14^. Similarly detergents also caused water pollution. Hence, the adsorption of dyes, pharmaceuticals, and detergents could be a possible solution to reduce water pollution. At first, the adsorption experiments were done using DS powder. Solutions of MB dye (22 mg/L) and ciprofloxacin (15 mg/L) in deionized water were prepared in the experiment. 20 ml of each MB dye and ciprofloxacin solution was taken in two separate beakers. In each beaker, 20 mg of DS powder was added. During adsorption, the stirring was provided continuously using magnetic stirrer. Test samples of 1 ml each was withdrawn from each beaker after every 5 min. The absorbance of test samples was measured using UV-visible spectrophotometer (SHIMADZU- UV 2600).The degradation in maximum absorbance value of dye and ciprofloxacin solution with respect to time is a direct quantification of adsorption capacity of DS powder. Similarly, during adsorption of detergent using DS powder, 0.25 g of detergent was taken in 100 ml of water. The quantification of adsorption of detergent using DS powder was done by noting down the values of pH values of test samples collected during adsorption experiment at the regular interval of 5 min.

In order to investigate the adsorption capacity of uncoated and DS coated non-woven fabrics, four petri dishes were taken. A 30 ml of MB solution was taken in two petri dishes separately. A 30 mL of ciprofloaxacin solution was taken in remaining two petri dishes separately. The uncoated and DS coated fabrics were dipped separately in two petri dishes containing MB dye solution. Similarly, the uncoated and DS coated fabrics were dipped separately in two petri dishes containing ciprofloxacin solution. The adsorption experiments were performed in dark and stirring was continuously provided by using magnetic stirrer. The quantification of adsorbance was done as given for DS powder case. In order to investigate the effect of number of layers on adsorption of MB dye, the adsorption experiment was repeated with two layers (total area of 34.4 cm^2^) and three layers (total area of 51.6 cm^2^) of DS coated non-woven fabric. In order to investigate the repeated performance of DS coated non-woven fabric, 10 cycles of adsorption-desorption of MB dye using three layers of DS coated non-woven fabric were performed. Desorption was done in acetone medium. The adsorption of detergent water was also performed using uncoated and DS coated non-woven fabric. As no adsorption peak was observed in case of detergent water in UV-visible spectrophotometer, so the quantification of absorption capacity using UV-visible spectrophotometer was not possible in this case. Two alternate ways were used: one was measuring pH value and other was measuring contact angle of detergent solution with time during adsorption experiment. The change in pH value and contact angle gave the direct measure of absorption of detergents. Each adsorption experiment was repeated four time in order to get mean and standard deviation (error) values.

## Supplementary information

Oil-water separation using uncoated non-woven fabric

Oil-water separation using DS coated non-woven fabric

Adsorption experiment

Diesel soot coated non-woven fabric for oil-water separation and adsorption applications

## Data Availability

All data generated or analyzed during this study are included in this published article.
