# Supplementary material for: Diesel soot coated non-woven fabric for oil-water separation and adsorption applications
Source: Sci Rep. 2019 Jun 11;9:8503. doi: 10.1038/s41598-019-44920-x (PMC6560123; doi:10.1038/s41598-019-44920-x)
Supplement: Supplementary file 4 — Diesel soot coated non-woven fabric for oil-water separation and adsorption applications [file 41598_2019_44920_MOESM4_ESM.docx]

**­Diesel soot coated non-woven fabric for oil-water separation and adsorption applications**

Moolchand Sharma^1^, Gurpreet Singh^1,*^, Rahul Vaish

School of Engineering, Indian Institute of Technology Mandi, Mandi, Himachal Pradesh 175005, India

***Corresponding Author E-mail address:** [gurpreetsinghc9@gmail.com](mailto:rahul@iitmandi.ac.in)

**Phone:** +91-1905-267138

**^1^Both authors have equally contributed**


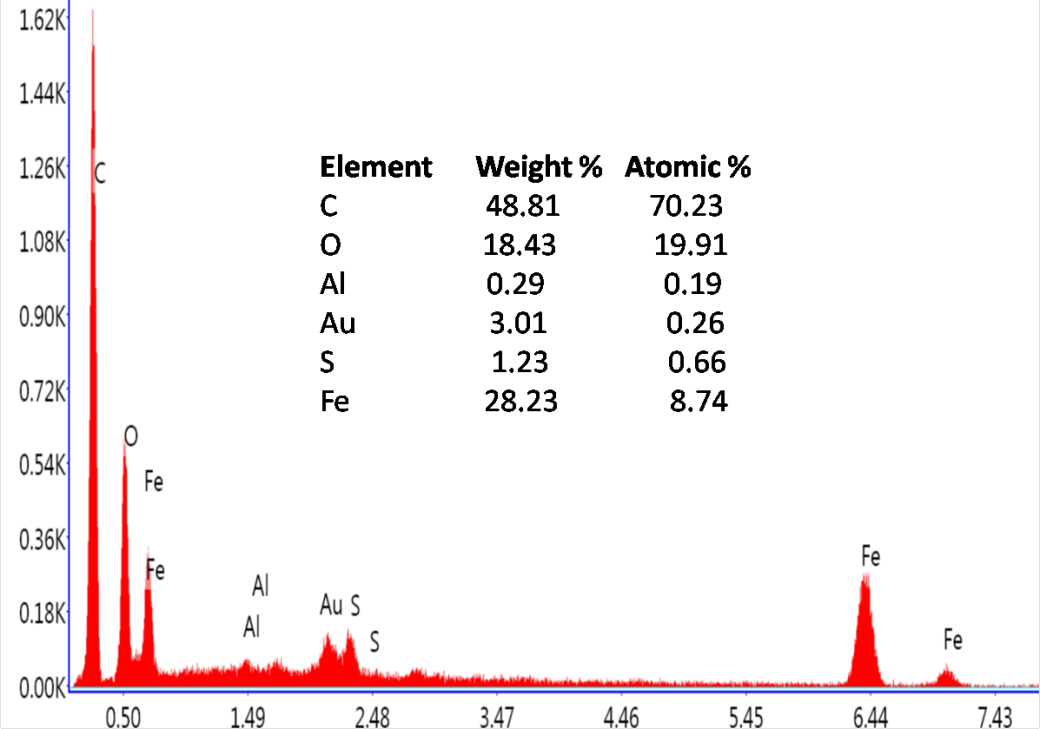


**Fig. 1. EDS spectrum of diesel soot (powder).**


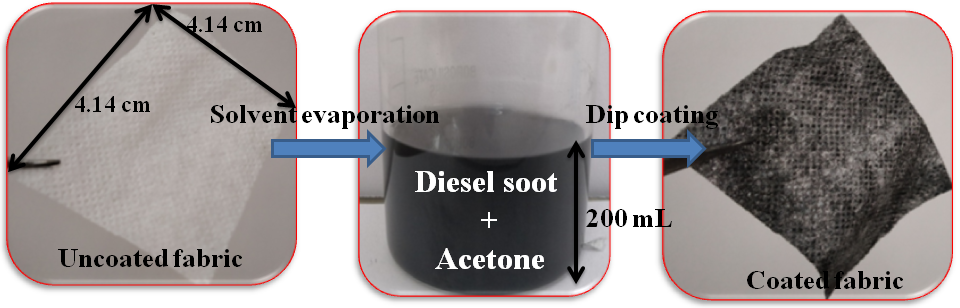


**Fig. 2. Schematic of fabrication of DS coated non-woven fabric.**


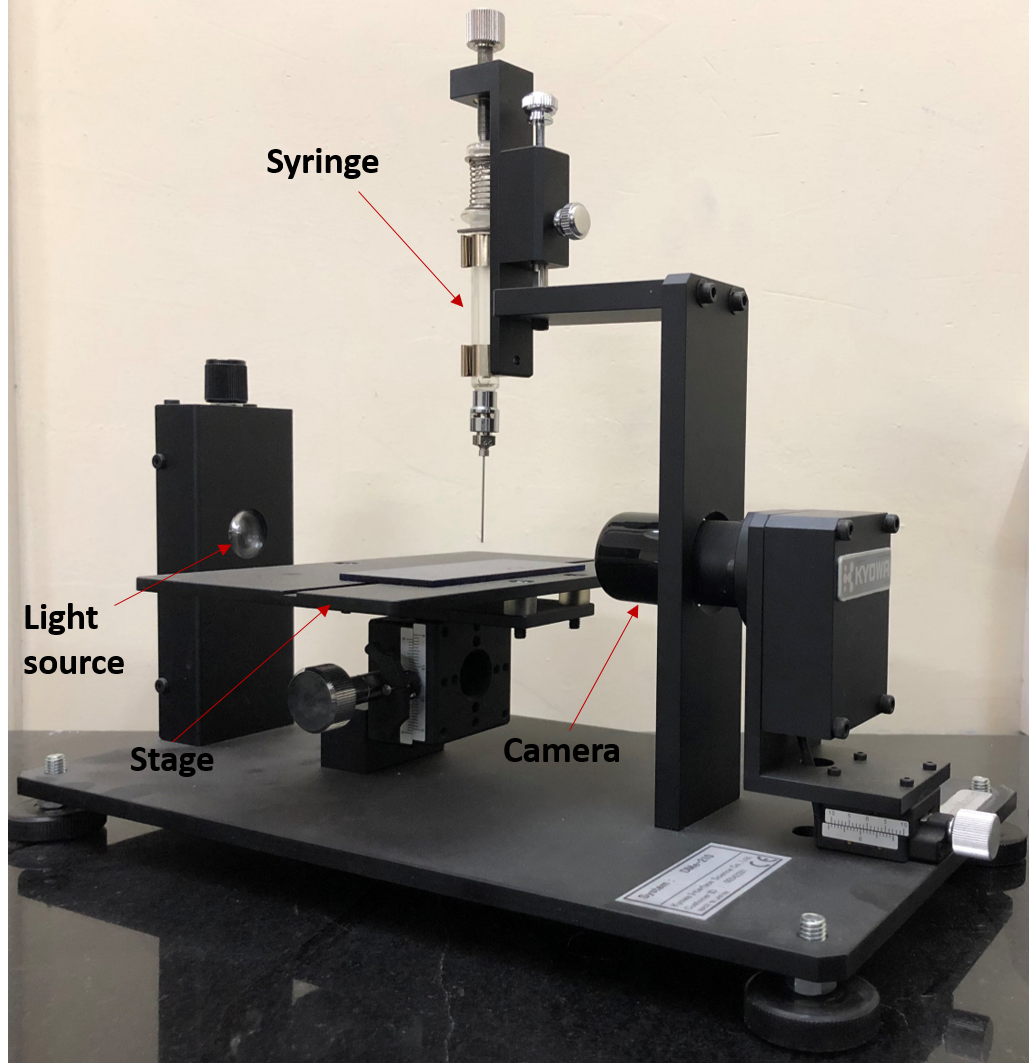


**Fig. 3. Contact angle meter (Kyowa Interface Science Co. Ltd., Japan).**

**Important details about contact angle meter:**

**Manufacturer:** Kyowa Interface Science Co. Ltd., Japan

**Camera:** CMOS camera: 30fps at 640$\times$480 pixels

**Motion of stage**: Stage (on which the sample is placed) can be moved manually only in vertical direction.

One of the important feature of this contact angle meter is that it provides **live image display** and **droplet volume monitoring**.

**Software**: An automated software named ''FAMAS Software Version 5.0.30" is installed on computer attached to contact angle meter. This software directly provides the value of contact angles on the screen. So, no manual calculation is needed.

**Table 1: Densities and viscosities of different oil at room temperature used in the present study.**

| Oil | Density (kg/m^3^) | Viscosity (cSt) |
| --- | --- | --- |
| Petrol | 682 [1] | 0.44 [1] |
| Diesel | 820 [1] | 2.87 [1] |
| Bean oil | 920 [2] | 41.4 [2] |
| Mustard oil | 925.24 [3] | 63.40 [3] |

[1] Chaichan, M. T. (2014). Combustion and emissions characteristics for DI diesel engine run by partially-premixed (PPCI) low temperature combustion (LTC) mode. *International Journal of Mechanical Engineering (IIJME)*, *2*(10), 7-16.

[2] Sagiroglu, A., Ozcan, H. M., Isbilir, S. S., Paluzar, H., & Toprakkiran, N. M. (2013). Alkali Catalysis of Different Vegetable Oils for Comparisons of Their Biodiesel Productivity. *Journal of Sustainable Bioenergy Systems*, *3*(01), 79.

[3] Azad, A. K., Uddin, S. A., & Alam, M. M. (2012). a comprehensive study of DI diesel engine performance withvegetable oil: an alternative bio-fuel source of energy. *International Journal of Automotive and Mechanical Engineering (IJAME)*, *5*, 576-586.





**Fig. 4. Absorbance vs concentration plot of MB dye solution.**

According to Beer-Lambert law," the absorbance is proportional to the concentration of the substance in the solution." Therefore, UV-visible spectroscopy can be used to measure the unknown concentration of dye in the dye solution.

Fig. 4 shows absorbance vs concentration plot of MB dye solution. The measured absorbance values of MB dye solution of various initial concentrations (10, 20, 30, 40, 50 mg/L) showed straight line. This clearly indicate that absorption and concentration of dye in solution are directly related to each other.

This clearly showed that the decrease in absorbance value directly indicated the decrease in concentration of dye in dye solution.
